# Canadian Consumer Preferences Regarding Gene-Edited Food Products

**DOI:** 10.3389/fgeed.2022.854334

**Published:** 2022-04-11

**Authors:** Oswaldo Vasquez, Hayley Hesseln, Stuart J. Smyth

**Affiliations:** Agricultural & Resource Economics, University of Saskatchewan, Saskatoon, SK, Canada

**Keywords:** food safety, genetic self-knowledge, innovation, neophobia, trust in government, willingness to consume

## Abstract

Innovations in food production and processing have largely remained “behind the scenes” for decades. The current nature of social media and calls for increased transparency regarding food results in a new landscape where consumer product demands are more important than ever, but are increasingly based on limited, or incorrect, information. One area where consumer awareness is rapidly emerging is the area of gene-edited food products. This article uses a consumer survey to gather perceptions regarding food safety, gene editing and willingness to consume for three gene-edited food products. Four factors were found to strongly influence consumer perceptions: trust in the Canadian food safety system; food technology neophobia scores; knowledge of genetics; and self-knowledge of gene editing. The survey of 497 Canadians found that 15% identified as neophobics and 12% as neophilics. The majority of participants identified as neutral. When presented with various food values, participants indicated that nutrition, price, and taste were the three most important values. A participants’ willingness to consume gene-edited food products strongly correlated with neophobic and neophilic preferences, with neophobics unwilling to consume and neophilics being uncertain. The only food value that strongly affects consumer willingness to consume is the environmental impact of a products’ production. Canadian consumers have a moderate to high level of trust in Canada’s food safety system, but this level of trust fails to carry over to food products produced through innovative technologies; however, consumers express a higher level of trust in gene-edited technology than genetically modified technology.

## 1 Introduction

Food is perhaps the most unique of products in terms of innovation acceptance, as there is no alternative to food capable of sustaining life. Virtually all other innovations have alternatives, allowing consumers who do not want to adopt a new innovation the opportunity to continue with their livelihood in a largely unaffected manner (e.g., microwaves, cellphones, autonomous driving vehicles). Consumers who express uncertainties about innovations within the production and processing of new foods and food products are restricted in alternatives, as labelling of attributes can be limited by space available and ability to quantify. The function of information is becoming increasingly essential to the adoption of innovations within the food industry.

One aspect of food innovation that has dramatically improved the innovative capacity of food production is the ability to alter a plant’s genetic makeup, initially through genetic modification, but more recently through gene editing (Smyth 2019). Research on genetic modification of plants began in the late 1980s, with the first varieties of genetically modified (GM) canola, corn, cotton and soy being commercialized in the mid-1990s. These initial varieties had traits inserted for herbicide tolerance and insect resistance, which provided significant producer benefits, but little in the way of consumer benefits (Klümper and Qaim 2014). These four crops are used as ingredients in a wide number of processed food products. Consumer perceptions of GM food products have been well documented as largely being concerned about their consumption (Gaskell et al., 2010; Prati et al., 2012; Frewer et al., 2013; Lusk et al., 2015; Vecchione et al., 2015; McFadden and Lusk 2016; Lusk et al., 2018).

Rapid adoption of gene editing technologies began in the first part of the 21st century. This was especially the case following the discovery of a new gene editing technology, CRISPR/cas9 (clustered regularly interspaced short palindromic repeats) (Doudna and Charpentier 2014). Gene editing technologies involve targeted and controlled changes of specific gene(s), rather than the insertion of foreign genetic material. Recent studies on consumer perceptions of gene editing technology and food products reveal that consumers have significant knowledge gaps about gene editing (Ishii and Araki 2016; McFadden and Smyth 2019; Sutherland et al., 2020a; Busch et al., 2021).

While some literature exists on consumer perceptions and attitudes regarding potential gene-edited (GEd) products (Tanaka 2017; Shew et al., 2018; Gatica-Arias et al., 2019), not much is known about consumer perceptions pertaining to GEd products currently available in the market. Many variables influence consumer attitudes toward food including personal tastes, risk concerns, and benefit perceptions, preferences for technologies used to produce food, and trust in federal food regulatory agencies (Hudson et al., 2015; The Strategic Counsel 2016; Sutherland et al., 2020b). The literature establishes that Canadian consumers have high levels of trust in Canadian food regulatory agencies (Hobbs and Goddard 2015; Health Canada 2016; Sutherland et al., 2020b), while possessing (self-admittedly) little to no understanding of gene editing technology.

The policy dilemma created by this high level of trust in existing food products, but uncertainty about the commercial availability of new GEd products, leaves the food industry questioning how best to communicate the safety of these products to consumers. With the scientific principles of developing GM and GEd crops being different, given that no foreign DNA is inserted in most GEd varieties, extrapolating consumer perspectives towards GEd food products based on GM food product surveys and experiments, can provide false impressions of GEd technologies. GEd crop technologies offer increased potential to enhance traits that provide direct benefits to consumers, such as enhanced nutrition (Xiao et al., 2020).

To address these gaps, this study uses survey data to identify drivers affecting Canadian’s perceptions of GEd food. The article examines factors affecting perceptions of food produced using gene editing techniques paying close attention to preferences based on the Food Technology Neophobia Scale (FTNS), and willingness to consume (WTC) three GEd food products—milk, apples, and potatoes. Gene-edited apples and potatoes are already on the market and GEd milk is a hypothetical product as GEd cattle are presently only used for research purposes.

### 1.1 Consumer Perceptions of Gene Editing

Gene editing technologies hold great promise for agriculture, food security, and food processing (Georges and Ray 2017). Previous plant breeding technologies, such as chemical or radiation mutagenesis, resulted in random, uncontrolled gene mutation, while the application of new breeding technologies (NBTs) such as CRISPR/Cas9, is a targeted, site-specific mutation technology. Estimates indicate that genes can be edited in a matter of days for as little as €10, with the development of new varieties available in 2–3 years: a significant reduction from the present 7–25 years (Friedrichs et al., 2019). While there are several GEd NBTs capable of utilization in variety development, CRISPR/Cas9 has exhibited the greatest potential and adoption to date (Lassoued et al., 2018; Eriksson et al., 2019). In late 2020, Japan approved the commercialization of a GEd tomato that has enhanced amino acids that are linked to lower blood pressure (Maxwell 2021), while in 2021, Japan also approved two GEd fish that require less feed to reach market (Nature Biotechnology 2021). Busch et al. (2021) undertook an assessment of perceptions regarding GEd in five countries (Austria, Canada, Germany, Italy, United States) finding that respondents believed the discussion should be less about whether it is correct to use this technology, but rather where it is appropriate to be used.

Lucht (2015) examined consumer attitudes regarding NBTs, suggesting that researchers and seed companies should consider this technology might encourage positive consumer perceptions. Tanaka (2017) explored the psychological factors affecting NBT product acceptance in Japan, finding consumer attitudes toward NBT-derived crops were more positive than their attitudes toward GM crops, when a definition of NBT was provided to participants. The results show perceived risks, perceived benefits, trust and bioethics influence NBT product acceptance.

Shew et al. (2018) conducted a multi-country assessment of willingness to pay and WTC using a hypothetical CRISPR rice compared to a GM rice involving respondents from the United States, Canada, Belgium, France, and Australia. Respondents were willing to consume both GM and CRISPR foods in the United States (56%), Canada (47%), Belgium (46%), France (30%), and Australia (51%). Participants were more willing to consume foods produced with CRISPR rather than GM. Familiarity with GM technology had a positive impact on the WTC in Australia (CRISPR and GM), Canada and the US (GM), and France (CRISPR). The main drivers of WTC CRISPR and GM were perceptions of safety, environmental attitudes, technology familiarity, and previous experience.

Gatica-Arias et al. (2019) analyzed perceptions and attitudes regarding the production and potential consumption of CRISPR/Cas9 crops in Costa Rica. In spite of very low knowledge or awareness of GEd crops or foods, respondents indicated preferences for GEd food products if nutrition was improved (71%) or prices were lower than other products (61%). Kato-Nitta et al. (2019) analyzed expert and public attitudes toward GEd crops compared to GM and conventional breeding in Japan. Results revealed that scientific knowledge influences consumer risk, benefits, and value perceptions. Public respondents had more positive attitudes toward GEd products than GM products. Muringai et al. (2020) used random utility models to analyze consumer recognition values of GM and GEd potato attributes. They found discounted prices were required for the purchase of GM and GEd potatoes, however, consumers were willing to pay more for a health attribute (reduced acrylamide) compared to environmental benefits.

### 1.2 Factors Affecting Consumer Perceptions

Cox and Evans (2008) developed a psychometric tool to identify levels of neophobia in relation to technology, which is based on the food neophobia scale first developed by (Pliner and Hobden, 1992). The FTNS is recognized as a valid and accurate tool for assessing consumer fear by focusing on the food technology rather than the food product (Henriques et al., 2009; Evans et al., 2010; Matin et al., 2012; Goddard et al., 2018). Yang and Hobbs (2020) analyzed information framing effects in consumer perception among GEd (CRISPR-Cas9). Their study compared the effectiveness of using logical-scientific versus narrative information to communicate with consumers about novel technologies in an online survey of 804 Canadian participants. Results showed that narratives help reduce negative perceptions regarding agricultural and food technologies.

Evans et al. (2010) validated the score for several novel food technologies such as pasteurization, fortification, and bioactives, while Cattaneo et al. (2019), used the FTNS to predict consumer behavioural attitudes to food that made use of novel technologies such as genetic modification, nanotechnology and modified atmosphere packaging. Lusk and Briggeman, 2009 found that individuals’ food choices could be explained by their preference for more abstract food quality attributes, known as “food values”, including naturalness, taste, price, safety, convenience, nutrition, tradition, origin, fairness, appearance, and environmental impact. Food values have been proposed as a method of identifying stable constructs of consumer preferences (Lister et al., 2017). Bazzani et al. (2018) compare food values in Norway and the United States, finding that the set of food values can explain individuals’ food choices across a variety of food products and does not depend on the specific context under investigation.

The majority of previous studies have assessed consumer preferences for GM food products, in a general attitude, rather than in reference to specific grocery store shelf products. The survey utilized in this article provides insights into consumer preferences for two GEd products beginning to appear in grocery stores, apples, and potatoes. The third product used is milk from a GEd cow, which at this point is a hypothetical product, given that GEd dairy cows are presently undergoing risk assessment, prior to receiving commercial approval (Molteni 2020).

## 2 Methodology

A consumer survey was conducted in August 2018 across english-speaking Canada using the University of Saskatchewan’s Social Sciences Research Laboratories, which contacted a pool of respondents administrated by EKOS Research Associates. Focusing on GEd and GM products, data were collected to evaluate consumer perceptions, willingness to consume, and the factors affecting the willingness to consume such products.

The survey explained the purpose of the research and sought information in three sections. First, respondents were asked to identify sources of information about food products; their beliefs of the most important food attributes (e.g., naturalness, price, safety); confidence in Canada’s food safety system; the degree of trust in organizations regarding food safety; and food technology neophobia statements (based on Matin and Goddard, 2013; Vidigal et al., 2015; Goddard et al., 2018).

To identify basic knowledge of food production, survey respondents completed a quiz of ten basic questions concerning genetics (Huang et al., 2006; Bett et al., 2010; Zhang et al., 2010; Vecchione et al., 2015; McFadden and Lusk 2016). Respondents were given the options: true, false, or don’t know. Correct answers were given one mark and minus one for each incorrect answer. Questions answered as don’t know received zero points. The variable “knowledge” is continuous between −10 (all questions answered incorrectly) and +10 (all questions answered correctly). Last, respondents rated their own knowledge about genetics and gene editing on a five-point scale from very poor to very good. Participants were not tested for knowledge prior to the survey.

To assess food neophobia scores, consumers were presented randomly with 13 statements (Table 1) and asked the extent to which they agreed on a seven-point Likert scale from 1 (strongly disagree) to 7 (strongly agree). The scores from these questions were used to classify whether a participant was neophobic, neutral or neophilic.

**TABLE 1:** **Food technology neophobia questions**.

New food technologies are something I am uncertain about
New foods are less healthy than traditional foods
The benefits of new food technologies are often grossly overstated
There are plenty of tasty foods around, so we do not need to use new food technologies to produce more
New food technologies decrease the natural quality of food
New food technologies are unlikely to have long-term negative health effects
New food technologies give people more control over their food choices
New products using new food technologies can help people have a balanced diet.
New food technologies have long-term negative environmental effects
It can be risky to switch to new food technologies too quickly
Society should not depend heavily on technologies to solve its food problems
There is no sense trying out high-tech food products because the ones I eat are already good enough
The media usually provides a balanced and unbiased view of new food technologies

The second section defined gene editing and how it differs from GM technologies. Participants were provided with definitions of gene editing and genetic modification, where GEd was defined as a plant breeding technique that modifies a plant’s genetic makeup, through enhancing, deleting, or altering specific parts of the DNA sequence. The GM process was defined by the difference that it involves the introduction of genetic material from different species. Participants were questioned on their agreement with sixteen statements about gene editing to determine perceptions based on: benefits, environmental risks, health risks, ethics, and equity [de Groote et al. (2016); Bett et al., 2010; de Groote et al. (2016); Kimenju and de Groote, 2005]. Participants were also questioned about their attitudes toward genetic modification and gene editing and their willingness to consume GM and GEd food products. The last section gathered demographic information.

Because one of the main objectives of this study was to evaluate categorical factors that affect consumers’ perceptions of genome editing, we use an ordered logit model. We expect that consumer perceptions and willingness to consumer will be positively influenced by, H1: confidence in the food safety system, H2: knowledge of genetics, and H3: self-rated understanding of genome editing. We also expect that the degree to which consumers are neophilic (H4) will positively influence perceptions.

Second, because attitudes based on a five-point Likert scale are used to establish differences between the variables that impact perceptions of each technology we use a multinomial logit model to determine consumers’ willingness to consume. Participants were questioned regarding their attitudes toward transgenics and genome editing and their WTC GM, genome-edited and organic food products. Specifically, we expect willingness to consume to be positively influenced by H5: perceived benefits, and negatively influenced by H6: environmental and health risks, ethical and equity concerns. The dependent variables of the multinomial logit model measure the WTC of three GEd food products: Innate potatoes^1^ and Arctic apples (both recently approved in Canada), and GEd milk (a hypothetical product). The benefits obtained by using gene editing technology are different for each product. Innate potatoes resist blackspot bruising and contain lower levels of asparagine; Arctic apples do not brown; and GEd milk is produced by dairy cows that have been GEd to be hornless. Horn removal is a stressful livestock treatment, and gene editing dairy cows to no longer have to endure this process, improves their welfare.

The same regressors are used in both regressions in order to establish differences between the variables that impact perceptions of each food technology. All qualitative data were coded and evaluated using descriptive statistics with Stata and Microsoft Excel and analyzed. The University of Saskatchewan Behavioural Research Ethics Board approved the survey (BEH #76). The survey dataset is not publicly accessible or available as this would violate the Research Code of Conduct at the University of Saskatchewan.

Independent variable categories were generated through the survey process (Supplementary Appendix S1A), which included food information sources, food values (e.g., Lusk and Briggeman, 2009), knowledge about genetics, and self-rated knowledge about genetics and gene editing, FTNS ratings, and demographic information. The FTNS aggregates responses to agree/disagree statements using a seven-point Likert scale. As Verneau et al. (2014) identify, the scale evaluates statements from four components. The first component, “new food technologies are unnecessary” measures feelings, worries about risks of new food technologies, uncertainty, adverse health effects and minimization of associated benefits. The second component, “perception risks” includes environmental, ideological, and risk perception for the evaluation of aversion to new food technologies. The third component evaluates “health benefit perceptions,” with the fourth focused on “information available in the media.” The FTNS ratings were derived through participant responses to 13 statements about new food technologies using a seven-point Likert scale ranging from strongly negative 1) to strongly positive 7) (Vidigal et al., 2015). Scores obtained allowed us to divide participants into three groups: neophilics who have a low level of food technology neophobia, and a strong affinity for novel food technologies; neutral respondents who have a medium level of food technology neophobia; and neophobic individuals who dislike new food technologies.

Benefit awareness and perceived risks regarding the environment, health, ethics, and equity were calculated using statements based on previous studies focused on consumers’ perceptions for GM food in Africa (Bett et al., 2010; Kimenju and de Groote, 2005; de Groote et al. (2016)). Original statements were adapted to obtain perception indices for gene editing technology.

Statements were shown to respondents in random order and grouped into five categories: benefits, environment risks, human health risks, ethical concerns, and equity concerns. Responses were given scores for their level of agreement: strongly disagree (−1), disagree (−0.5), neither agree nor disagree (0), agree (0.5), strongly agree 1) and category indices were used.

### 2.1 Socio-Demographics

A total of 503 individuals participated in the survey and after removing incomplete information, 497 observations remained. The survey sample closely reflects the Canadian english-speaking population with some slight differences. Sample data slightly over-represents those with higher-incomes and under-represents those in the lowest income categories, particularly $50,000 - $70,000, which accounted for 19% of the population in Canada but only represents 9% of survey respondents. The sample is also skewed towards higher-educated individuals, with about 52% having a bachelor or graduate degree. Because the survey was conducted in English, Quebec is under-represented with only one respondent. There was no representation from the Northwest Territories, Nunavut, or Yukon. Finally, fewer than 4% of participants reported working in an agri-food related field. Table 2 provides a comparison of survey respondents to the most recent Canadian census results.

**TABLE 2:** Socio-economic characteristics (*n* = 497).

	**Survey sample (%)**	**Canadian population (%)**
Age range	—	—
Under 25	4.99	8.22
25–34	18.36	16.91
35–44	16.37	16.65
45–54	20.76	18.46
55–64	19.76	18
Over 65	19.16	21.75
Gender	—	—
Male	46.1	49.11
Female	53.3	50.89
Annual household income (before taxes)	—	—
<$30,000	6.19	9.82
$30001–$50000	14.17	17.84
$50001–$70000	9.38	19.29
$70001–$90000	12.18	16.76
$90001–$150000	28.14	26.28
>$150000	13.37	10
Education	—	—
Graduate + bachelor’s degree	52.20	28.5
University below bachelors	8.38	3.1
College diploma	20.76	22.4
Apprenticeship or other trades certificate	4.99	10.8
High school diploma	10.38	23.7
No certificate diploma or degree	1.40	11.5

## 3 Results

**FIGURE 1:**
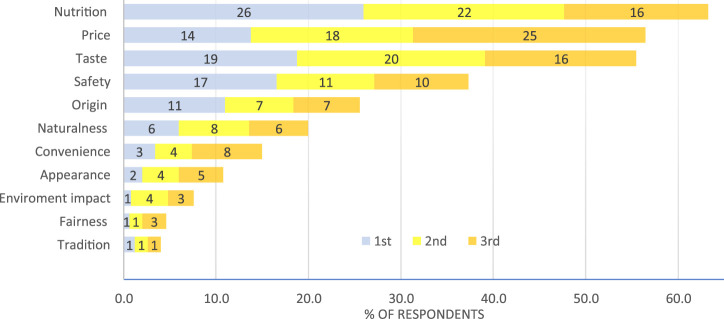
Top three food values. Note: Consumers were presented with a random list and asked to select the top three choices in terms of value of food choices. Nutrition (nutritional food quality), Price, Taste, Safety (confidence in Canada’s food safety system), Origin (where the food was grown or produced), Naturalness (the degree to which food has been altered), Convenience (of use), Appearance, Environmental impact (effect on biodiversity), Fairness (of production), Tradition (historical precedence).

Respondents were randomly presented with a list of 11 different food values, the results indicating that nutrition (63%), price (56%), and taste (57%) were the three most important, all scoring above 50%. Next was food safety under 40%, with the remainder scoring below 30% (Figure 1). Less than 5% of respondents considered tradition or fairness as their top choice. The results indicate how dominant nutrition, price, taste and to a lesser extent, safety, are for consumers, as their responses indicate the preference for food that is safe, nutritious, and cheap.

Regarding participants’ confidence in Canada’s food safety system about half (49%) were confident or very confident, 24% each were moderately confident and somewhat confident, with only 2.6% not at all confident. The levels of confidence are lower than those identified by Health Canada (2016) where 66% of Canadians express confidence in Canada’s food safety.

The food information providers that ranked highest in terms of trust were Health Canada (79%), the Canadian Food Inspection Agency (75%) and Agriculture and Agri-food Canada (67%). The three least trusted organizations were food processors (22%), retailers (18%), and animal welfare advocacy organizations (18%).

Findings regarding subject knowledge based on the 10-question quiz indicate that on average, participants chose the correct answer half of the time. One question was answered incorrectly for most of the participants: corn grown thousands of years ago looks the same as corn grown today (62% incorrect). There were three questions for which people were largely uncertain (don’t know): whether it is possible to transfer animal genes to plants (53%), the use of radiation to create genetic mutations (42%), and the possibility that catfish genes would alter the flavour of a tomato (41%).

Regarding previous knowledge about food technologies, more than half of respondents self-rated as very poor or poor for genetics (50%), plant breeding (56%) and gene editing (72%). Only 8% of participants self-rated as having a good or very good understanding of gene editing.

Results of the FTNS were defined from the sample average (47.28) plus and minus one standard deviation (10.75). Most participants fell into the neutral category at 73%, followed by neophobics at 15%, with only 12% registering as neophilics.

Benefit and risk perception indices are illustrated in Table 3. The majority of participants provided positive responses for perceived benefits (agree and strongly agree) with a score of 0.28, which is the highest index across the five evaluated criteria, while less than 7% chose negative responses (disagree and strongly disagree). The perceived benefits responses align with the preferences for nutritious and safe food identified in Figure 1. Results showed a considerable level of concern with environmental risks, specifically, potential loss of original plant varieties (50%). The results highlight inconsistencies as only 17% of respondents agreed that GEd crops would have negative environmental impacts, yet 50% agreed that GEd crops would have a negative impact on biodiversity. However, responses to the other two statements indicated lower levels of concern. The general perceived environmental risks index is 0.14.

**TABLE 3:** Benefits and risk perception indices toward gene editing technology (%ages).

		Statement	Agree/strongly agree	Neither agree nor disagree/don’t know	Disagree/strongly disagree	Perception score
Perceived benefits	1	Gene editing technology has the potential to create foods with enhanced nutritional value	**56.28**	37.13	6.59	0.3
	2	Gene editing has the potential to reduce pesticide residue on food	**49.1**	45.71	5.19	0.26
	3	Gene editing has the potential to reduce pesticide residue in the environment	45.31	49.7	4.99	0.25
	4	Gene editing technology can result in insect-resistant crops	**53.29**	42.52	4.19	0.31
		Benefits perception index	—	—	—	**0.28**
	5	Gene-edited crops are negative for the environment	17.17	57.28	25.55	-0.03
Perceived environmental risks	6	Insect-resistant crops developed using gene editing could cause death of untargeted insects	38.32	54.29	7.39	0.2
	7	Gene editing can lead to a loss of original plant varieties	**49.7**	37.32	12.98	0.24
		Environmental perception index	—	—	—	**0.14**
Perceived health risks	8	Consuming gene-edited food products can damage human health	19.56	55.49	24.95	-0.02
	9	Consuming gene-edited foods products can lead to more allergies	20.96	62.08	16.96	0.04
	10	Consuming gene-edited foods might lead to an increase in antibiotic-resistant diseases	26.15	58.48	15.37	0.06
		Health risk perception index	—	—	—	**0.03**
Ethical concerns	11	Gene editing is tampering with nature	**55.69**	27.75	16.56	0.24
	12	Gene editing technology makers are imitating God	21.76	40.12	38.13	-0.13
	13	Gene-edited food is not natural	**46.11**	36.92	16.97	0.19
		Ethical perception index	—	—	—	**0.10**
Equity concerns	14	Gene-edited products only benefit multinational producers	23.75	48.31	27.94	0
	15	Gene-edited products don’t benefit smaller farms	24.75	49.9	25.35	0.02
	16	Gene-edited products are being forced on developing countries by developed countries	17.96	65.87	16.17	0.02
		Equity concerns perception index	—	—	—	**0.01**

Note: bolded numbers are significant at the 95% confidence level.

The health risk index is 0.03. Respondents’ uncertainty is noticeably higher in this category as those indicating they neither agreed or disagreed was the dominant response option. Response inconsistencies are again present as 25% disagreed that GEd crops could harm human health, yet 26% agreed that GEd crops could lead to a rise in antibiotic-resistance diseases.

From an ethical perspective, more than half of respondents agree that the use of GM is tampering with nature, and that genetically modified food is not natural (46%). The number of genes in a plant ranges from hundreds of thousands to millions, depending on the species and the rate of natural mutation from one generation of a plant to the next, ranges from 10^−5^–10^−6^ in corn (Stadler 1930) to 10^−9^ in arabidopsis (Kovalchuk et al., 2000). This contributes to the confirmation that the public lacks knowledge regarding the science of plant breeding and the notion of genes changing due to science is not natural. This can be further reinforced by the result mentioned above, where 62% of respondents incorrectly believe that modern day corn looks identical to corn that was produced several thousand years ago. However, scores for “neither agree nor disagree” are slightly higher resulting in the ethical index being 0.1.

Responses between agree and disagree are relatively evenly split in the equity section, with the largest spread between the two positions being 4%. The majority of responses indicated they neither agreed nor disagreed.

Prior to reporting attitudes toward GM and gene editing, participants were provided with a statement highlighting the main differences between the two. Results revealed that the majority of respondents expressed a neutral attitude towards both: GM (54%) and gene editing (50%). With respect to a positive attitude results show a greater positive attitude (positive and strongly positive) for gene editing (29%) in comparison to GM (16%). The reverse was true for negative and strongly negative attitudes.

### 3.1 Regression Results

#### 3.1.1 Consumer Perceptions of Gene Editing and GM Technology

The dependent variables of the ordered logit model (OLM) regressions measure the attitudes toward gene editing and GM based on a five-point Likert scale ranging from strongly negative 1) to strongly positive 5). The model used 66 variables, of which eight were statistically significant for gene editing perceptions and nine for GM.

McFadden’s Pseudo R^2^ was calculated to provide a parameter to register goodness of fit for the model. In the case of the ordered logit model, the R^2^ of gene editing and GM consumer perceptions is equal to 0.16 and 0.18, which means that the models explain only 16 and 18% of the variance in the dependent variables. The estimated coefficients of perception of gene editing and GM from the ordered logit regression are expressed in Table 4. The table shows only the coefficients that are statistically significant. For GEd, being neophilic or neophobic influenced attitudes as did trust in Canada’s food safety system (not at all confident and very confident), and self-rated understanding (all categories but poor). Attitudes to GM products were influenced by convenience and social media, being neophobic or neophilic, being confident in Canada’s food safety system, and having a self-rated understanding of gene editing for all categories but very good.

**TABLE 4:** Parameters estimated for Ordered Logit Model.

Variables	GEd	GM
Convenience ranked top 3	--	0.789** (0.327)
Neophilic	0.956*** (0.293)	1.189*** (0.288)
Neophobic	−1.05*** (0.337)	−0.911** (0.362)
Social media	--	0.499** (0.239)
Trust in Canada’s food safety system		--
Not at all confident	−1.985** (0.808)	
Very confident	0.760** (0.333)	0.647 (0.333)
Self-rated understanding gene editing		−0.95 ** (0.368)
Very poor	−1.05*** (0.351)	
Poor	--	−0.676** (0.311)
Very good	2.244** (0.978)	--
Knowledge	0.158*** (0.043)	0.093** (0.041)
Gender	0.676*** (0.212)	0.378* (0.217)

Statistical significance at 1% (***) and 5% (**) levels. Standard error in parentheses.

Note: The table reports only significant parameter estimates of each regression, with (--) indicating that results were not significant. Full model estimation results are available upon request.

Two categorical variables relating to the level of confidence in Canada’s food safety system (not at all confident and very confident) are statistically significant (5%) indicating that consumers who are not at all confident in the Canadian food safety system tend to have a negative perception of GEd technology. Alternatively, respondents who felt very confident tend to have a positive perception of GEd technology. The categories “confident” and “very confident” had only a small impact on the perception of GM technology. Results for FTNS were significant with expected signs for both GM and GEd technologies. Neophilic consumers tend to have positive perceptions of technology whereas neophobic consumers had negative perceptions.

Knowledge about basic genetics has a positive impact on the perception of GEd and GM technology. The greater the degree of self-rated understanding, the greater the likelihood of acceptance, with the opposite also being significant.

#### 3.1.2 Willingness to Consume Gene-Edited Food Products—Multinomial Logit Model

The dependent variables relate to three categories defining whether a respondent would consume food produced using GEd technology: yes, no, and don’t know.

The model used the same variables as in the OLM regressions, except for the addition of the five perception index variables. McFadden’s Pseudo R^2^ was also calculated to register goodness of fit: 0.39 for the GEd potato; 0.34 for the GEd apples; and 0.27 for GEd milk. Results explain 39, 34 and 27% of the response variance.

Statistically significant coefficients are expressed in Table 5. Consumers who were not sure of whether they would consume GEd potatoes were neophilic and chose food origin (where the food was grown or produced) as part of their top three food values. Respondents who responded negatively to consumption also ranked environmental impacts as one of the top three concerns and saw perceived health risks associated with GEd technology. Finally, individuals more likely to consume GEd potatoes had a high level of trust in the Canadian food safety system and perceived benefits associated with GEd production. According to (Rodríguez-Entrena and Salazar-Ordóñez, 2015) public-perceived risk play a critical factor in determining acceptance of novel food technology. The other perception indices did not have significant impacts on WTC.

**TABLE 5:** Parameters estimated from Multinomial Logit Model.

Variables	Gene-edited potato		Gene-edited apple		Gene-edited milk	
	Yes	No	Yes	No	Yes	No
Trust in Canada’s Food Safety System	0.083	0.437	0.396	0.985**	0.876**	0.863**
(Not at all Confident)	(0.419)	(0.472)	(0.437)	(0.453)	(0.389)	(0.387)
Neophilic (food neophobia score)	−1.379***	−2.059***	−0.820*	−0.505	−0.765	−0.306
	(0.467)	(0.740)	(0.480)	(0.551)	(0.434)	(0.488)
Neophobic (food neophobia score)	−0.499	−0.011	0.758	1.408**	−1.423**	0.116
	(0.620)	(0.577)	(0.727)	(0.688)	(0.694)	(0.474)
Food origin (ranked in top three concerns)	−1.086**	−0.927	−0.956	−0.561	−0.249	−0.350
	(0.479)	(0.525)	(0.503)	(0.521)	(0.446)	(0.424)
Environmental impact (ranked in top three concerns)	1.047	2.171***	1.180	2.645***	0.573	1.020*
	(0.743)	(0.758)	(0.834)	(0.807)	(0.666)	(0.596)
Friend or family (source of information)	−0.027	−0.084	0.287	0.200	0.687**	0.200
	(0.320)	(0.372)	(0.334)	(0.351)	(0.305)	(0.295)
Government website (source of information)	0.867**	0.677	0.583	0.353	0.268	-0.072
	(0.428)	(0.483)	(0.429)	(0.452)	(0.371)	(0.372)
Food company (source of information)	−0.340	−1.325**	−0.300	−0.749	0.224	0.115
	(0.432)	(0.488)	(0.433)	(0.449)	(0.369)	(0.369)
Self-rated understanding of gene editing	−0.531	−0.567	−0.215	−0.030	−1.657***	−0.918
(very poor)	(0.595)	(0.669)	(0.605)	(0.610)	(0.567)	(0.536)
Benefits perception index	2.774***	−1.022	2.512***	0.957	1.715***	−0.077
	(0.653)	(0.808)	(0.668)	(0.702)	(0.563)	(0.576)
Health risk perception index	0.360	1.880**	−1.091	0.234	−0.508	0.601
	(0.786)	(0.931)	(0.852)	(0.909)	(0.702)	(0.739)
Ethical perception index	−0.957	1.082	−0.297	0.298	−0.058	1.114**
	(0.558)	(0.675)	(0.579)	(0.639)	(0.506)	(0.533)
Gender	0.138	−1.300***	−0.316	−1.46***	−0.310	−0.652**
	(0.337	(0.405)	(0.351)	(0.378)	(0.317)	(0.315)

Note: Statistical significance at 1% (***) and 5% (**). Standard error in parentheses. The table reports only significant parameter estimates.

The WTC of GEd apples is impacted by both food technology neophobia categories. Nevertheless only “neophobic” is strongly significant. Results show that neophobics are not willing to consume GEd apples whereas neophilics tend to respond, ‘don’t know.’ Environmental impact is the only food value that strongly affects the WTC indicating that respondents who valued the environment are less likely to consume apples relative to having a neutral position. Similar to the GEd potatoes, this is likely due to the identification of environmental risk associated with the production of GEd food products in general, consistent with Bett et al. (2010) and de Groote et al. (2016).

Finally, having a positive benefit perception results in a greater likelihood in WTC. The impact from benefit perception indices among novel food consumption were also identified by Bett et al. (2010) and de Groote et al. (2016). However, regression results also show a negative effect on WTC from participants who do not trust at all Canada’s safety system.

Willingness to consume GEd milk, is positively associated with a high level of trust in Canada’s food safety system. Being neophobic is also is statistically significant with the expected sign: respondents were less likely to consume. Low neophobia does not have a strong impact on WTC GEd milk, however neophilics, unexpectedly, are more likely to respond, “don’t know” rather than “yes.” Perceiving benefits from GEd technology is positively associated with WTC, while perceiving ethics risks does the opposite.

## 4 Discussion

Canadian respondents to this survey present a paradox. Respondents indicate they are highly confident in Canada’s food safety system, which aligns with previous studies (Hobbs and Goddard 2015; Health Canada 2016; Sutherland et al., 2020b). Yet the majority identify they know little about the science of food production, resulting in low trust levels in food products created using innovative technologies, which also aligns with previous studies (Ishii and Araki 2016; McFadden and Smyth 2019; Sutherland et al., 2020a; Busch et al., 2021). Respondents expressed strong confidence in the same system that would ensure the safety of new, innovative food products, so, why is there such a confidence gap?

Respondents self-identified as having low levels of knowledge regarding plant breeding technologies that are used to develop new crops and ultimately food products, corroborating the findings of Sutherland et al. (2020a). The lack of knowledge creates uncertainties about new food products, resulting in consumers’ aversions to purchasing such products. This was strongly evident with those identifying as neophobic. Yet, nearly three-quarters of respondents identify as neither neophobic or neophilic, indicating that they desire further information regarding new food products, prior to rejecting or supporting these products. Other studies, such as Yang and Hobbs (2020), identify that narratives aid in decision making and the lack of narratives in this study is anticipated to have contributed to respondents identifying as neither neophobic or neophilic.

Perceptions about gene editing technology is varied as identified in Table 3. Support by those who strongly or somewhat strongly agree that GEd crops could provide enhanced nutrition, reduced pesticide residues in food and the environment and produce insect-resistant crops ranged from 45 to 56%, while those who strongly or somewhat disagreed ranged from 4 to 7%. The dichotomy is evident where the results indicate that 45% of respondents agree or strongly agree that GEd crops can reduce pesticide residues in the environment, yet 50% agree or strongly agree that GEd crops can reduce the number of original plant varieties, or biodiversity. Respondents have the perspective that GEd crops can produce an environmental benefit through fewer pesticide residues, but also harm the environment, by lowering biodiversity. These results are in line with McFadden and Smyth (2019) who reported the majority of Canadians believe modern plant breeding will increase production as well as lead to a loss of biodiversity. For a review of the benefits of genome editing technologies, (Smyth, 2021).

The lack of knowledge about the basic principles of genetics and gene mutation is highlighted in the result that 56% of respondents agree or strongly agree that gene editing is viewed as “tampering” with nature. This finding is reinforced by the 62% of respondents that incorrectly indicated that corn cobs of today are virtually unchanged from those of several thousand years previous. These results align with those of Busch et al. (2021), who found the majority of respondents opposed to the use of GEd technologies, perceived that GEd was “tampering” with nature. Awareness that genes in any species are capable of mutating from one generation to the next is not part of common public knowledge, hence, consumers are expressing uncertainties and reservations about new products that simply incorporate aspects of nature. As identified above, gene editing techniques allow for precision and the ability to control individual gene mutation. Gene mutation is part of the natural evolution of plants and in some instances, the induced mutation from gene editing could have less of an effect on a plant’s overall genetic sequence than the rate of natural mutation.

Based on the results of this survey, the gap in confidence expressed by consumers between the safety of the current food system in Canada and new, innovative products entering into Canadian food markets, is largely the result of a lack of knowledge or awareness of basic genetics. Without knowledge that plants, and every other species, will naturally experience genetic mutation, the effort and investment to provide this level of knowledge will be substantial and lengthy.

## 5 Conclusion

Results of this research indicate that Canadian consumers have a more positive perspective about gene editing technology as compared to GM technology. Trust in the Canadian food safety system and self-rated understanding of gene editing, as well as being neophobic point to the importance of the availability of scientific and reliable information for consumers about emerging food technology. The research finds that surveyed Canadians have low levels of scientific knowledge, and a low level of self-rated knowledge of gene editing technology, which limits the consumption intention for GEd food products. There is, therefore, a definite need for better scientific disclosure to educate consumers about gene editing technology. According to Lucht (2015), educated consumers with objective information will tend to weigh risks and benefits in a rational way. However, it is important to consider that a strategic campaign based exclusively on the information deficit model could lead consumers to a confirmation bias. A more appropriate approach would consider ideological beliefs, and consider the public’s ethical, political, religious, and culture views. Since the FTNS was identified as an important driver of respondents’ willingness to consume GEd food products, consumer characteristics should be considered in the design of communication strategies. Canadian consumers were strongly affected by two factors: risks and health characteristics (Chen et al., 2013). Therefore, to offset food technology neophobia, education campaigns must inform consumers about GEd food products in terms of health risks and consumption.

Findings from the consumer survey also revealed that most Canadians believe there are benefits to gene editing technology, particularly with respect to nutrition, and reduction of pesticide residues in food and in the environment. Consumers also identified benefits from gene editing technology. The perceived benefits play a significant role in the WTC of GEd food products; therefore, information campaigns should be focused on strengthening consumers’ already positive perceptions about nutritional contributions and pest-resistant characteristics. According to Gatica-Arias et al. (2019), low levels of knowledge about gene editing occur because information generated in scientific studies has not been communicated effectively to consumers. On the other hand, the WTC of GEd food products could be negatively affected by perceptions about environmental effects, particularly the loss of original plant varieties. Concerns related to unnaturalness, untrustworthiness, uncertainty, unhealthiness, and risks are frequently associated with GM production (Chen et al., 2013) and consumers tend to consider gene editing as similar to GM (Kato-Nitta et al., 2019), therefore, it is also important to highlight the differences with GM technology to avoid and prevent misconceptions of emerging novel food technologies.

Considering the important impact that Canada’s food safety system has on consumer perceptions of gene editing technology, institutions play a fundamental role as information providers to consumers. According to Lucht (2015), consumers have limited knowledge and depend on entities they consider trustworthy to make informed decisions. Therefore, Health Canada, the Canadian Food Inspection Agency, and Agriculture and Agri-food Canada are likely the most appropriate information dissemination channels for consumer studies and regulation standards status. The use of modern media sources to disseminate scientific knowledge is of crucial importance for effectively communicating biotechnology findings to the public (Wunderlich and Gatto, 2015), therefore the inclusion of interactive media should be considered for the introduction of GEd food products into the Canadian market.

## Data Availability

The raw data supporting the conclusion of this article will be made available by the authors, without undue reservation.

## Footnotes

1 While the Canadian Food Inspection Agency has regulated Innate potatoes as plants with novel traits, J. R. Simplot identifies that reduced oxidation has been achieved by genome editing. For details, see: https://www.innatepotatoes.com/technology.
