# Supplementary material for: Canadian Consumer Preferences Regarding Gene-Edited Food Products
Source: Front Genome Ed. 2022 Apr 11;4:854334. doi: 10.3389/fgeed.2022.854334 (PMC9035513; doi:10.3389/fgeed.2022.854334)
Supplement: Supplementary file 1 [file Table1.pdf]

Appendix A – Summary Statistics and Results

Independent Variables	Description
Classic sources (radio, TV, magazines, newspaper)	Dummy, 1 if radio, TV, magazines are sources used t access information about food products and 0 otherwise
Family/friends	Dummy, 1 if family / friends are a source used to acces information about food products and 0 otherwise.
Scientific sources (conferences, agronomist, professional / scientific publication)	Dummy, 1 if conferences, agronomist services c scientific publications are a source used to acces information about food products and 0 otherwise.
Social media (Facebook, Twitter, Snapchat, Instagram)	Dummy, 1 if social media is a source used to acces information about food products and 0 otherwise.
Company (food company website, food label)	Dummy, 1 if information provided from food companie are a source to access information about food produc and 0 otherwise.
Government institution websites	Dummy, 1 if official websites are a source to acces information about food products and 0 otherwise.
Naturalness ranked top three	Dummy, 1 if naturalness was ranked top 3 and otherwise
Taste ranked top three	Dummy, 1 if taste was ranked top 3 and 0 otherwise
Price ranked top three	Dummy, 1 if price was ranked top 3 and 0 otherwise
Safety ranked top three	Dummy, 1 if safety was ranked top 3 and 0 otherwise
Convenience ranked top three	Dummy, 1 if convenience was ranked top 3 and otherwise
Tradition ranked top three	Dummy, 1 if tradition was ranked top 3 and 0 otherwise
Origin ranked top three	Dummy, 1 if origin was ranked top 3 and 0 otherwise
Fairness ranked top three	Dummy, 1 if fairness was ranked top 3 and 0 otherwise
Appearance ranked top three	Dummy, 1 if appearance was ranked top 3 and otherwise
Environment impact ranked top three	Dummy, 1 if environment was ranked top 3 and otherwise
Trust in Canada's food safety system (four variables)	Categorial variable (1=not at all confident, 2=somewha confident, 4= confident, 5=very confident)
Knowledge	Knowledge score
Self-rated understanding genetics	Categorial variable (1=very poor, 2=poor, 4= goo 5=very good)
Self-rated understanding genome editing	Categorial variable (1=very poor, 2=poor, 4= goo 5=very good)
Neophilico	Binary, 1 if individual is neophilico and 0 if neutral
Neophobic	Binary, 1 if individual is neophobic and 0 if neutral
Gender	Dummy (1 = male)
